# Pathological changes in a patient with acute respiratory distress syndrome and H7N9 influenza virus infection

**DOI:** 10.1186/s13054-014-0666-y

**Published:** 2014-12-05

**Authors:** Qiang Guo, Jian-an Huang, Daguo Zhao, Jun Jin, Shenlang Liu, Dustin R Fraidenburg

**Affiliations:** Department of Medicine, Respiratory, Emergency and Intensive Care Medicine, The First Affiliated Hospital of Soochow University, 188 Shizhi Street, Suzhou, 215006 China; Department of Medicine, Division of Pulmonary, Critical Care, Sleep and Allergy Medicine, University of Illinois at Chicago, Chicago, IL 60612 USA

There are very few data regarding pathological changes in patients with severe pneumonia and acute respiratory distress syndrome (ARDS) from avian influenza A (H7N9) virus. We present the case of a 73-year-old woman with a history of hypertension and prior cerebrovascular accident who was admitted to the hospital complaining of dyspnea, cough with scant hemoptysis, and fever. A history of live poultry exposure was elicited during an initial interview.

Hypoxic respiratory failure led to endotracheal intubation and mechanical ventilation on the first day of hospitalization. Significant lymphopenia was also noted upon admission. Infection with influenza A (H7N9) virus was confirmed from a tracheal aspirate sample using polymerase chain reaction assays. Thoracic imaging revealed diffuse bilateral infiltrates that were associated with impaired gas exchange and a diagnosis of ARDS. Despite the administration of oseltamivir and peramivir, the patient continued to deteriorate. Bronchoscopy with bronchoalveolar lavage specimens suggested possible co-infection with four potential pathogens (*Pseudomonas aeruginosa*, *Flavobacterium meringosepticum*, *Staphylococcus haemolyticus*, and *Candida albicans*) and empiric antimicrobial therapy was begun.

Computed tomography revealed ground glass opacities and consolidation in both lungs. High positive end-expiratory pressure (>7 cmH_2_O, 27 days total) and high plateau pressures (>30 cmH_2_O, 27 days total) were necessary as rescue therapies in order to maintain the goal of partial pressure of arterial oxygen >55 mmHg. Mechanical ventilation was complicated by right pneumothorax on day 9, and chest tube drainage was performed that resulted in a persistent air leak. Severe sepsis and ARDS was complicated by stress cardiomyopathy and the patient expired on hospital day 27.

A limited autopsy was performed and small samples of right upper lung tissue were obtained, fixed, and embedded. The samples were used for hematoxylin and eosin staining, Masson staining, and smooth muscle actin immunohistochemistry staining. Histopathological evaluations revealed diffuse alveolar damage characterized by edema, hyaline membranes, inflammation, and fibrosis. Fibrin thrombus within the vascular lumen, inflammatory infiltrate below the endothelium, and necrosis of bronchiolar walls were also present (Figure 1). It is notable that these findings show similarity to the pattern observed in patients of avian influenza (H5N1) [1,2] and 2009 influenza A (H1N1) [3,4]. Immunohistochemistry showed severe fibrosis and thickened distal pulmonary arteries in the samples obtained (Figure 1).

**Figure 1:**
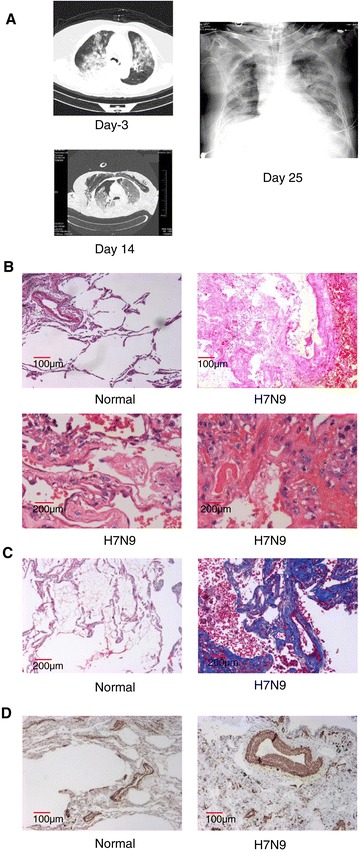
**Micrographs of lung tissue, pathophysiology, and treatment from a patient with acute respiratory distress syndrome and H7N9 influenza. (A)** Two computed tomography images (3 days before admission (day –3) and 14 days after admission (day 14)) and the patient’s last X-ray image (2 days before death (day 25)). **(B)** Hematoxylin and eosin staining from a normal sample and the H7N9 patient’s lungs. Diffuse alveolar damage, including edema, hyaline membranes, and inflammation, were seen in these images. **(C)** Masson staining in normal lungs and the H7N9 patient’s lungs. Severe fibrosis (blue) was seen in the H7N9 patient. **(D)** Immunohistochemical staining with smooth muscle actin in normal lungs and the H7N9 patient’s lungs. Thickened distal pulmonary arteries were seen in the H7N9 patient.

This case illustrates that, in addition to the primary viral pneumonia, complications including ARDS and bacterial co-infections may contribute to diffuse alveolar damage, severe fibrosis, and thickened distal pulmonary arteries as the most significant and consistent finding observed with severe H7N9 virus infection. More studies are needed to further characterize the pathogenesis and to determine effective treatment strategies for severe respiratory illness caused by H7N9 virus infection.

## Abbreviation

ARDS: Acute respiratory distress syndrome
